# Enhanced Room-Temperature Thermoelectric Performance of 2D-SnSe Alloys via Electric-Current-Assisted Sintering

**DOI:** 10.3390/ma16020509

**Published:** 2023-01-05

**Authors:** Kesavan Manibalan, Meng-Yuan Ho, You-Cheng Du, Hung-Wei Chen, Hsin-Jay Wu

**Affiliations:** Department of Materials Science and Engineering, National Yang Ming Chiao Tung University, Hsinchu 30010, Taiwan

**Keywords:** thermoelectrics, SnSe single crystal, 2D materials, radio frequency sputtering, multistep deposition

## Abstract

Single-crystalline tin-selenide (SnSe) has emerged as a high-performance and eco-friendly alternative to the lead-chalcogens often used in mid-temperature thermoelectric (TE) generators. At high temperature >800 K, the phase transition from *Pnma* to *Cmcm* causes a significant rise in the TE figure-of-merit (*zT*) curve. Conversely, the SnSe TE requires a booster at low temperatures, which allows broader applicability from a device perspective. Herein, a synergy of Cu alloy and Ag-coating is realized through a sequential multi-step synthesis, designed to combine different metal deposition effects. Single-crystalline (Cu_2_Se)*_x_*(SnSe)_1−*x*_ alloys grown by the Bridgman method were then coated with a thin Ag layer by radio frequency (RF) sputtering, and the interlayer epitaxial film was observed via electric-current assisted sintering (ECAS). Consequently, the thin Ag-coating improves the electrical conductivity (*σ*) and reduces the thermal conductivity (*κ*) for (Cu_2_Se)_0.005_(SnSe)_0.995_+Ag alloy, increasing the *zT* curve at close to room temperature (373 K). The incorporation of multistep addition by ECAS enables tuning of the overall solubility of the alloy, which opens a new avenue to optimize TE performance in anisotropic 2D materials.

## 1. Introduction

The emerging low-carbon economy is promoting high-performance renewable energy aimed at reducing the carbon footprint of energy production and consumption. A thermoelectric (TE) material converts unused waste heat into electricity through the Seebeck effect [1], evolving as a promising low-carbon energy resource. TE materials produce electricity that can be measured by a dimensionless “figure of merit”, (*zT*) = (*S*^2^/*ρκ*) T, where *S*, *ρ*, and *κ* are Seebeck coefficient, electrical conductivity, and thermal conductivity, respectively. The *zT* formula describes the interplay between electrical transport and thermal conduction. The correlation of *S*, *ρ*, and *κ* leads to a complex decoupling of the power factor (*PF*) = *S*^2^/*ρ* from *κ*. Recently, anisotropic two-dimensional (2D) materials have arisen as promising TE candidates, because they exhibit high efficiency in *PF-κ* cleavage [2]. The reported SnSe single crystal reaches *zT* index > 2 above 800 K for its *Cmcm* phase that performs a high in-plane *PF* of 8 μWcm^−1^K^−2^ with an intrinsically low *κ* of 0.4 Wm^−1^K^−1^ [3,4]. Although the out-of-plane *κ* (along the *a*-axis) can reach the amorphous limit of SnSe [5], the layered structure hinders electrical transport and *PF* [6]. The low-temperature *Pnma* SnSe phase displays a relatively low-lying *σ* (high *ρ*) with a high *S*, reflecting its intrinsically low hole carrier density (*n_H_*) of 10^17^ cm^−3^ [7].

Alkali metals are added to SnSe single crystals, which improves the *zT* values in the 300–800 K temperature range [8]. Despite their excellent TE performance, hole-reduced SnSe single crystals have a narrow optimization window and reproducibility. Aside from that, the layered structure of SnSe single crystals poses an obstacle to the mass production and assembly of TE due to weakly confined inter-plane cleavage [9]. Contrary to the SnSe polycrystals, the rapid and inexpensive synthesis increases their mechanical strength [10,11]. Nevertheless, SnSe polycrystals generate lower *zT* due to the reduced *σ*. Texturing and doping SnSe polycrystals with Cu [12,13], Ag [14,15], Al [16], and Pb [17], etc., leads to enhanced *σ* and reduced *κ*, inducing SnSe-based high-performance alloys with adequate mechanical strength. Particularly when Cu_2_Se is used, superionic Cu^+^ can be kinetically disordered by Se sublattices’ elicit liquid-like behavior, which eliminates shear stress vibrations, resulting in low lattice thermal conductivity (*κ_L_*) and high *PF*. As a heavier element, Ag has both a larger atomic radius (134 Å) and atomic mass (108) than Cu (117 Å, 63.5), suggesting that Ag addition can cause extra point defects, leading to mass and strain field fluctuations that promote a further decrease in *κ* and increase in *PF*. The performance improvements resulting from the incorporation of Cu and Ag into SnSe crystals have significant implications for the development of new alloys [14,15,18].

Following the benefit of metal incorporation in improving SnSe TE performance, this work sequentially incorporates Cu and Ag into SnSe single crystal alloys. The Bridgman method is used to alloy SnSe and Cu_2_Se. Thin layer Ag-coating is then performed by RF sputtering, followed by the ECAS process. A two-step approach is used to deposit Cu and Ag that accommodate different metal insertion solubility. Notably, at near room temperature, co-deposited (Cu_2_Se)_0.005_(SnSe)_0.995_ + Ag alloys have a higher *zT* value than pristine SnSe single crystals [4] and polycrystals [13]. As a result, the synergy of incorporating co-metals via multi-step synthesis improves SnSe TE performance, transforming the 2D material applicability into a green and cost-effective TE coolant.

## 2. Experimental

### 2.1. Bridgman Crystal Growth

High purity elements, Sn (99.99%), Se (99.99%), and Cu (99.99%), were used. Three important stoichiometric proportions were weighed, as indicated in Table 1, then they were placed in a conical bottom quartz ampoule (Ø12.5 mm) and sealed after evacuation (∼10^−3^ Pa). The ampoule was then inserted into a Bridgman reactor, heated to 1373 K for 5 h, and annealed at 1223 K for 10 h (reduction rate 1.5 mmh^−1^). After cooling, the obtained (Cu_2_Se)*_x_*(SnSe)_1−*x*_ bulk ingot was cut into pieces for further use.

### 2.2. Ag Sputtering

**Figure 1:**
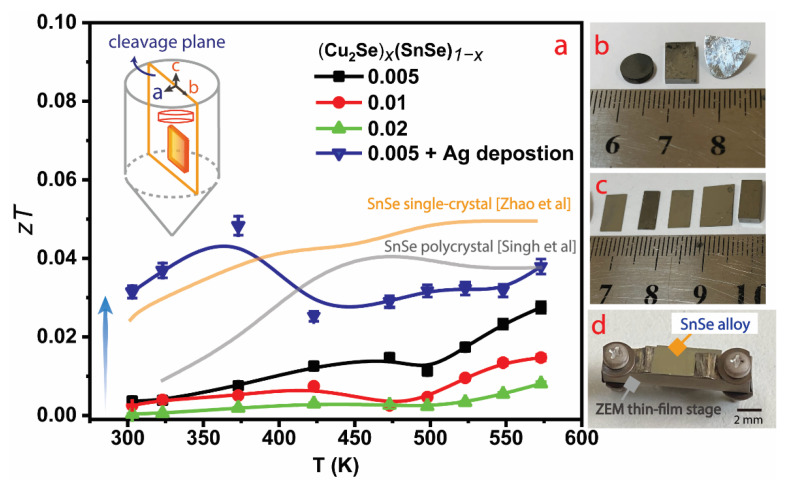
(**a**) Temperature-dependent *zT* profiles for (Cu_2_Se)*_x_*(SnSe)_1−*x*_ (*x* = 0.005, 0.01 and 0.02) and Ag-coating. The (Cu_2_Se)_0.005_(SnSe)_0.995_ + Ag alloy is denoted as a solid curve, and reported SnSe single-crystal [4] and polycrystal [13] are added for comparison. Inset: Pictographic of TE properties measurement along the in-plane direction (*bc* plane). (**b**) Left to right: Crystalline image for *κ* measurement (left, disk-shaped pellet), *PF* measurement (middle), cleavage plane from the as-grown bulk SnSe (right). (**c**) Plate-shaped SnSe alloys are sliced up to measure transport properties. (**d**) SnSe alloys were placed on a ZEM thin-film stage commercial apparatus for *S* and *σ* measurement.

RF sputtering was processed using a homemade Rex 300 instrument. The sputtered samples were then sintered by the ECAS method. In detail, the target was mounted on a metal plate, then fixed into the evacuation chamber and evacuated (10^−3^ Torr). After that, a cryogenic vacuum (10^−3^ torr) was provided for 1 h under Ar flow (15 SCCM). After heating to 100 °C, Ag sputtering (35W) was supplied for 1 h, resulting in Ag-sputtered (Cu_2_Se)_0.005_(SnSe)_0.995_ + Ag with a thickness of ~0.5 mm. The samples were cut and polished in a specific direction (Figure 1a) to measure the in-plane electrical and thermal transport properties of the samples.

### 2.3. Characterization

The crystal structure was examined by in-house powder X-ray diffraction (Bruker D2-Phaser, Ettlingen, Germany) with Cu K*α* radiation (*λ* = 1.5406 Å). X-ray photoelectron spectroscopy (XPS, ESCA, PHI 5000 Versaprobe II, ULVAC, Chigasaki, Japan) was used to determine the valence states of the (Cu_2_Se)_0.005_(SnSe)_0.995_ + Ag alloy. Morphological analysis was studied with a high-resolution transmission electron microscope (HR-TEM, JEOL JEM-F200, Tokyo, Japan), and EDS mapping was conducted using a Super X EDS detector.

### 2.4. TE Measurement

The *S* and *σ* were measured from 300 to 575 K in a thin helium atmosphere using a ZEM instrument (ULVAC-RIKO, ZEM-3, Yokohama, Japan). The relation of *κ* = DCpd, density (d) was measured by the Archimedes method, and thermal diffusivity (D) was measured using laser flash instruments (LFA 467, Netzsch, Berlin, Germany). Specific heat (Cp) was calculated by the Dulong–Petit law Cp (3R/M). Hall coefficient RH, *n_H_*, and carrier mobility (*μ*_H_) of the (Cu_2_Se)*_x_*(SnSe)_1*-x*_ + Ag alloy were measured with a Hall measurement system (HMS-3000) under a reversible magnetic field (0.49T) with 10 mA current.

## 3. Results and Discussion

The extremely mobile Cu ions in Cu_2_Se [19] and the SnSe lone pair electrons [7] induce an innately low *κ_L_* with better TE performance. An alloying strategy with dilute Cu_2_Se emerges rationally to enhance the SnSe TE efficiency in the low-temperature range [19]. For this purpose, three SnSe alloys were grown with dilute Cu_2_Se by the Bridgman method according to the stoichiometric composition (Cu_2_Se)*_x_*(SnSe)_1−*x*_ (*x* = 0.005, 0.01, and 0.02). The X-ray diffraction patterns of the best-performing control alloy and its nominal Ag-coating of (Cu_2_Se)_0.005_(SnSe)_0.995_ and (Cu_2_Se)_0.005_(SnSe)_0.995_ +Ag at low temperature were indexed (Figure S1). According to previously observed patterns [13,20] Cu doped SnSe formed an intrinsic Cu_2_Se secondary phase with sharp intensity peaks at 25°, 27°, 29°, 31°, 47°, and 65°, correlating to (201), (111), (011), (400), (511), and (800) as (Cu_2_Se)_0.005_(SnSe)_0.995_ alloy (Figure S1a,c). In the interim, a secondary phase of AgSnSe_2_ emerged during Ag sputtered in the (Cu_2_Se)*_x_*(SnSe)_1−*x*_ matrix during the SPS process, indicating the Ag presents in the bulk matrix based on preexisting models. Hence, Ag addition to (Cu_2_Se)_0.005_(SnSe)_0.995_ alloy, AgSnSe_2_, leads to impurity peaks at 43–44°, 55–56°, and 59°, attributed to (501), (312), and (420) crystal planes as (Cu_2_Se)_0.005_(SnSe)_0.995_ + Ag alloy (Figure S1b,d). This peak fit the XRD pattern of the previously reported impurity peak formation of the AgSnSe_2_ phase [21,22], demonstrating the sample’s purity. For SnSe-based alloys cut from the grown bulks (Figure 1b), transport characteristics along the in-plane direction (*bc* plane) were measured (Figure 1c). A custom thin film stage was employed to evaluate the in-plane *PF* of the alloy (Figure 1d). A disk-shaped pellet (Figure 1b) was also cut from the grown bulk to assess the in-plane *κ* over the temperature range of 300 K to 575 K. All x-series temperature-dependent *zT* curves can be computed from the average *PF* and *κ* values in triplicate (Figure 1a). The *x* = 0.005 performed relatively better than the other *x*-series alloys, reaching a *zT* of ~0.03 at 575 K (Figure 1a). To further improve the TE performance, the *x* = 0.005 alloy was incorporated with Ag via RF-sputtering, followed by ECAS. It can be seen that ECAS accelerates the epitaxial interlayer growth between sputtered Ag and the (Cu_2_Se)_0.005_(SnSe)_0.995_ matrix, and this enables Ag deposition in the (Cu_2_Se)_0.005_(SnSe)_0.995_+Ag alloy. As a result, the previously reported SnSe single crystals [4] and SnSe polycrystals [13] were greatly outperformed, with the *zT* curve rising to 0.0483 at 373 K.

**Figure 2:**
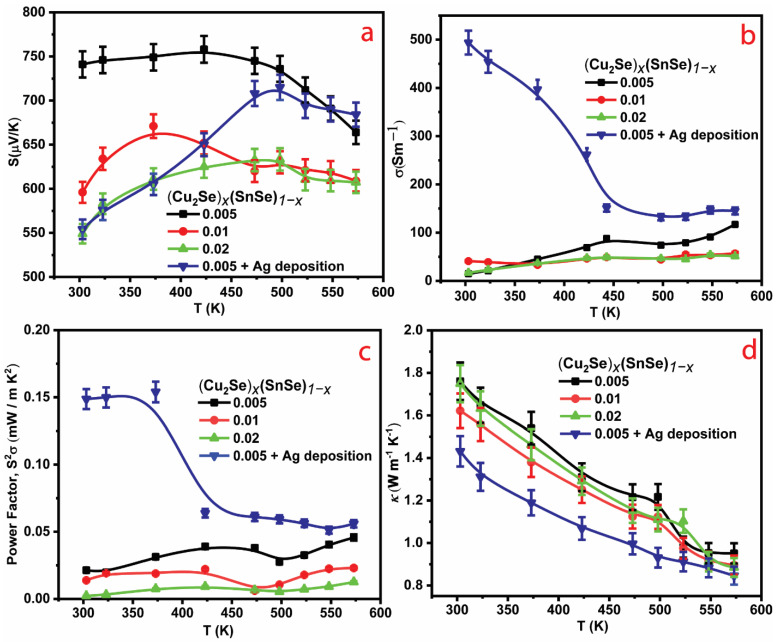
Temperature-dependent (**a**) Seebeck coefficient, *S*, (**b**) electrical conductivity, *σ*, (**c**) power factor, *S^2^σ***,** (**d**) thermal conductivity, *κ* of (Cu_2_Se)*_x_*(SnSe)_1_−*_x_*+Ag (*x* = 0.005, 0.01, and 0.02) alloys.

The temperature-dependent *S*, *σ*, *PF,* and *κ* curves with the average of three repeated analysis for all bare alloys and the Ag-coated (Cu_2_Se)_0.005_(SnSe)_0.995_ alloy are summarized in Figure 2. For bare alloys, both the *S* and *σ* curves exhibit a descending trend with increasing *x* (Figure 2a,b). According to theory, an optimum *n_H_* value shall bring an increase in *S* and a decrease in *σ*, because acoustic phonons dominate the scattering in a metal or degenerate semiconductor [17,18]. The addition of Cu_2_Se in SnSe leads to superionic kinetic Cu^+^ diffusion into the Se sublattice, which can eliminate shear stress vibrations and achieve good TE properties. High Cu solubility in the SnSe alloy would stabilize the high *n_H_*, implying fewer cation vacancies. As a result of approaching *x* = 0.005, the maximum solubility resulted in average *κ* (1.76 W m^−1^ K^−1^) and *PF* (0.0231mW m^−1^ K^−2^), and high *S* (741 μV K^−1^) when compared to pure SnSe. However, due to the increased band gap, the *n*_H_ was reduced ($9.31\times 10^{15}{cm}^{-3})$ below the ideal range, which requires further optimization. After this, as the quantity of Cu_2_Se (*x* = 0.01, 0.02) increases, its effective mass also increases, resulting in a modest rise in *n_H_* ((1$.31\times 10^{16}{cm}^{-3},x=0.01$) and ($1.11\times 10^{16}{cm}^{-3},x=0.02))$, and exhibits almost identical *κ* (1.62 W m^−1^ K^−1^, *x* = 0.01 and 1.74 W m^−1^ K^−1^, *x* = 0.02). Due to the disordered Cu atoms during the α/β phase transition, its *PF*, *S*, and *σ* exhibit disordered TE competence. The superionic Cu^+^ atoms can be used to strengthen the Cu_2_Se lattices, and their additional mobility (*μ*_H_) affects the overall TE properties (Table 1). The best performing sample (*x* = 0.005) shows an optimal *n*_H_-*x* (carrier-cation) exchange, resulting in higher *μ*_H_ transport. The highly-mobile Cu ion presumably produces a high *μ*_H_ that enhances *σ* without affecting *S*. Accordingly, *x* = 0.005 shows an improved *PF* curve (Figure 2c) compared to the other alloys in the *x*-series, allowing further incorporation of metal (Ag).

**Figure 3:**
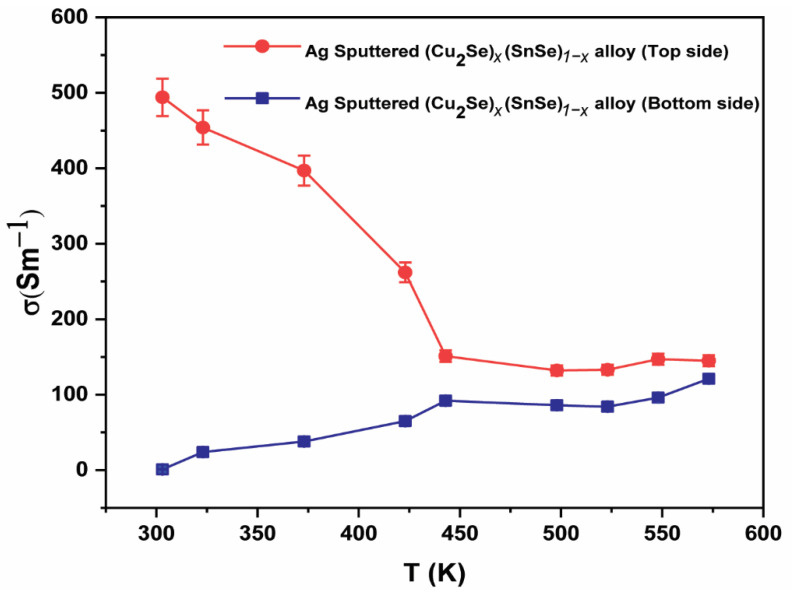
The electrical conductivity measured from both sides of the (Cu_2_Se)_0.005_(SnSe)_0.995_+Ag alloy (top and back/bottom sides).

As mentioned earlier, with a heavier element of Ag with a larger atomic radius and mass (134 Å, 108) than Cu (117 Å, 63.5), Ag-coating can lead to additional point defects and mass and strain-field fluctuations that further reduce *κ* and increase *PF*. Accordingly, a significant increase in the *zT* curve for the Ag-coated (Cu_2_Se)_0.005_(SnSe)_0.995_ alloy was obtained compared to the Ag-free alloy, and this augmentation was attributed to an optimally high *n*_H_ (${3.65\times 10}^{17}{cm}^{-3})$ and *μ*_H_ (149 cm^2^ V^−1^ s^−1^) that accounted for a larger *σ* (Figure 2b). In addition, the *PF* rises to ~0.149 mW/mK^2^ at 303 K and reaches a maximum of 0.154 mW/mK^2^ at 373 K, which is 15 times higher than the sample without an Ag-coating. Aside from the improved *PF*, the thin layer Ag-coating lowers the curve slightly (Figure 2d) due to enhanced phonon scattering, whereas high concentration causes irregularities and poor performance (*x* = 0.01, 0.02). If the same amount of Ag is added as in these systems, it may have a similar effect. Thus, coupling Ag with (Cu_2_Se)_0.005_(SnSe)_0.995_ leads to rational tuning of the band gap, *n_H_*, grain boundary, and *μ*_H_, which is more likely to contribute to high TE efficiency than TE pre-Ag coated samples. Therefore, the successive suppression of holes by the ECAS process realizes improved *PF* and reduced *κ* in 2D materials, opening a new pathway for *PF*-*κ* decoupling and *zT* optimization. Furthermore, the Ag layer is much thinner than the (Cu_2_Se)_0.005_(SnSe)_0.995_ alloy, and the accuracy of the measurement and coating effect on both sides of the alloy surface was confirmed using similar test conditions. Here, the *S* measurement was performed on the opposite side of the alloy (free of Ag film). Temperature dependent *σ* exhibits unequal values on both sides (top and bottom of the alloy) because the top part (top side) of the alloy is completely covered by the Ag layer, whereas the bottom part (bottom side) of the alloy cannot be covered by the Ag layer (Figure 3). Meanwhile, uncovered Ag layer of (Cu_2_Se)_0.005_(SnSe)_0.995_ disk *κ* measurement was not performed (bottom side), because Ag coated and uncoated (Cu_2_Se)_0.005_(SnSe)_0.995_ alloy may have produced nearly the same *κ* value (Table 1); thus, we decided to discontinue further investigation of the Ag uncovered portion of the alloy.

**Figure 4:**
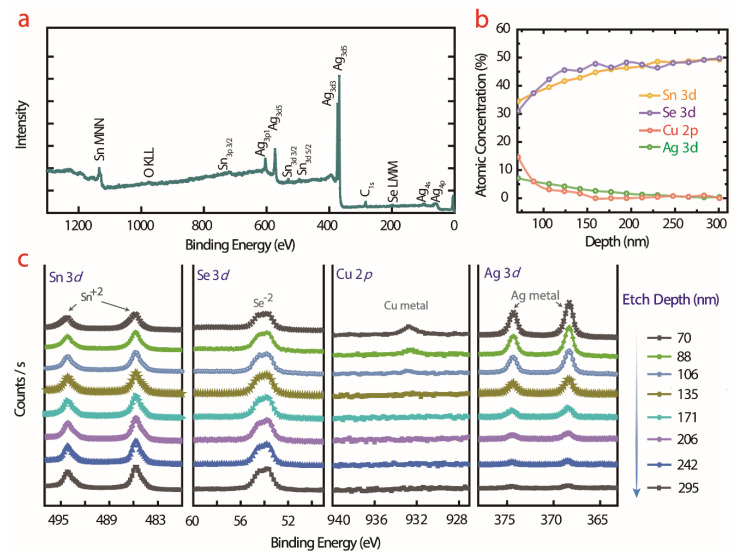
XPS analysis of (Cu_2_Se)_0.005_(SnSe)_0.995_ + Ag alloy. (**a**) Full surface survey spectrum, (**b**) depth profile, (**c**) high-resolution scan.

The XPS study reveals the mechanism behind the enhanced TE performance. To confirm the coexistence of incorporated Cu and Ag, the valence states of the (Cu_2_Se)_0.005_(SnSe)_0.995_ + Ag alloys were analyzed by XPS. The spectra show the entire binding energy range, indicating the occurrence of Sn, Se, Cu, and Ag energy states (Figure 4a). The XPS depth profile analysis revealed atomic composition ranges of Sn & Se > 45% and Cu & Ag < 5% (Figure 4b). The XPS spectra revealed spin-orbit doublet coupling of Sn 3d^5/2^ and Sn 3d^3/2^ appearing at the binding energies of 486.5 eV and 495.0 eV, respectively, stating the existence of Sn^2+^ ions in the alloy (Figure 4c). The spin-orbit coupling of Se 3d splitting shows a broad peak at 54.0 eV; this remains the same for both samples, indicating that Se^2−^ maintains its oxidation state. The substitution of Cu at the Sn site was verified by the occurrence of the 2p peaks at 933 eV. In addition, the coating of Ag doublet peaks at 367.5 eV and 374 eV is consistent with binding energies of 3d^5/2^ and 3d^3/2^, which is decisive in SnSe electronic properties and is in good agreement with the earlier report [13]. Thus, the measured atomic weights and concentrations of SnSe and deposited elements match well for ~0.3% of Sn vacancies.

**Figure 5:**
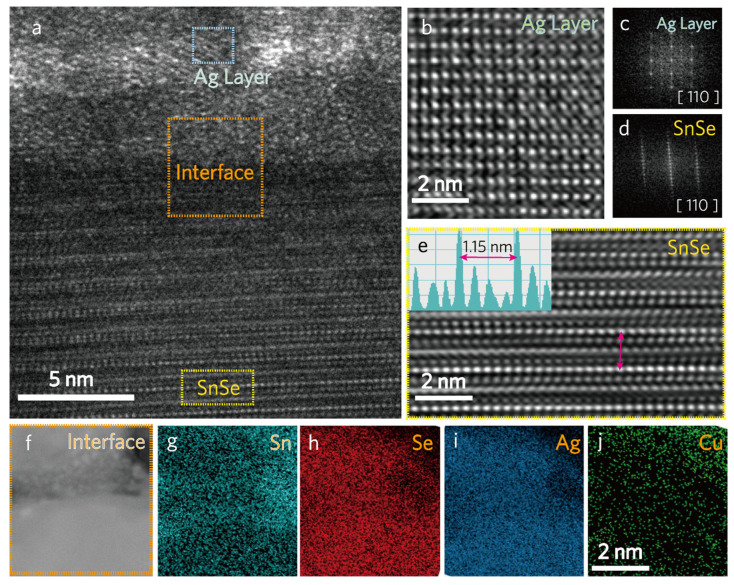
TEM analysis of (Cu_2_Se)_0.005_(SnSe)_0.995_+Ag alloy: (**a**) A cross-sectional BF image, (**b**) IFFT image of magnified Ag layer region (**c**,**d**) SAED pattern, (**e**) IIFT image of selected area lattice spacing’s, (**f**–**j**) Elemental mapping taken by EDX-STEM.

To understand the mechanism and origin of the low *κ_L_*, TEM analysis was performed on the best-performing (Cu_2_Se)_0.005_(SnSe)_0.995_ + Ag alloy. A bright field (BF) image shows the modular nanostructure of the sample (Figure 5a) with an epilayer-like Ag film topping feature on a (Cu_2_Se)_0.005_(SnSe)_0.995_ alloy. The dark contrasted areas are the Ag deposition, whereas the faint gray contrast corresponds to the (Cu_2_Se)_0.005_(SnSe)_0.995_ alloy. The high-resolution inverse FFT image displays the lattice arrangements of the (Cu_2_Se)_0.005_(SnSe)_0.995_ alloy (Figure 5b) and the sputtered Ag film (Figure 5e), while the corresponding selected area electron diffraction (SAED) patterns (Figure 5c,d) confirm an identical zone axis of (110) of both the Ag film and (Cu_2_Se)_0.005_(SnSe)_0.995_ substrate. The lattice spacing between the two apparent planes along the lateral dimension is estimated to be 1.15 nm, which reveals that a set of (110) planes stabilizes nanodomains (Figure 5e). Furthermore, the EDX elemental mapping performed during STEM imaging revealed several contrasting grain domains with a random distribution, confirming the presence of Ag, Cu, Sn, and Se within the (Cu_2_Se)_0.005_(SnSe)_0.995_ + Ag alloy (Figure 5f–j). From the results of TEM and STEM, the interface between the Ag film and (Cu_2_Se)_0.005_(SnSe)_0.995_ is semi-coherent and full of line dislocations as the lattice parameters between the matrix and the Ag film differ from each other. The interfacial mismatch and imperfections cause a significant reduction in *κ_L_* and a substantial increase in the point defect scattering of the resulting alloy (Table 1). Although the (Cu_2_Se)_0.005_(SnSe)_0.995_+Ag alloy has a bilayer structure, note that a trace of Ag can be seen from both the EDX element mapping (Figure 5i, Figures S2 and S3) and the XPS depth profile (Figure 4b). Sputtering between the Ag film and the SnSe substrate provides another way to attenuate holes and vacancies. Note that the (Cu_2_Se)_0.005_(SnSe)_0.995_+Ag alloy still exhibits interfacial inhomogeneity. The contact between the adjacent layers affects the accuracy of the reported TE transport properties. Therefore, future study will focus on each side of the uniform coating and explore them systematically. For comparison, the bilayer/interface layer-based SnSe alloy TE properties were tabulated (Table S1) and correlated to this present study, and the data revealed a relatively better TE performance than the previously reported method, which encourages further establishment.

## 4. Conclusions

In conclusion, a low-cost, low-temperature working green route TE alloy, (Cu_2_Se)*_x_*(SnSe)_1*-x*_ + Ag (*x* = 0, 0.005, 0.01, and 0.02), was synthesized by the Bridgman method, followed by the ECAS method. The in-plane *n_H_* of (Cu_2_Se)*_x_*(SnSe)_1-*x*_ was found to be 9.31×10^15^ after dilute addition of Cu_2_Se to SnSe (*x* = 0.005), despite its role contributing to a high *PF* with a modest *zT* of 0.03 at 573K. Excessive hole suppression and interlayer deposition were achieved by successful Ag-coating on the (Cu_2_Se)_0.005_(SnSe)_0.995_ alloy, which further promotes in-plane *n_H_* of 3.65 × 10^17^ with good TE performance. TEM analysis of the (Cu_2_Se)_0.005_(SnSe)_0.995_ + Ag alloy suggests a bilayer 2D nanostructured modular feature with an interlayer between the SnSe matrix and the sputtered Ag film. This interlayer accretion optimally scatters the heat-carrying phonons and results in a lower *κ*. Hence, the *zT* of (Cu_2_Se)_0.005_(SnSe)_0.995_ + Ag alloy at near room-temperature is 15 times higher than that of pristine SnSe (*zT* = 0.0483 at 373K). The incorporation of Cu and Ag in dilute form can be considered as an effective strategy for both *PF* enhancement and *κ_L_* reduction, thereby presenting a new key to improving *zT* of 2D TE materials.

## Figures and Tables

**Table 1:** Measured properties of (Cu_2_Se)*_x_*(SnSe)_1−*x*_ and Ag-doped alloy at 303 K.

(Cu_2_Se)*_x_*(SnSe)_1−*x*_	S (μV K^−1^)	*σ* (S m^−1^)	PF (mW m^−1^ K^−2^)	*κ* (W m^−1^ K^−1^)	*n* (cm^−3^)	μ (cm^2^ V^−1^ s^−1^)
*x* = 0.005	741	15	0.0231	1.76	9.31 × 10^15^	91.6
*x* = 0.01	596	41	0.01384	1.62	1.31 × 10^16^	77.6
*x* = 0.02	549	17	0.00224	1.74	1.11 × 10^16^	102.2
Ag-coated *x* = 0.005	554	494	0.1487	1.43	3.65 × 10^17^	149

## Supplementary Materials

The following supporting information can be downloaded at: https://www.mdpi.com/article/10.3390/ma16020509/s1. Refs. [23,24,25,26,27,28] are cited in the supplementary materials.
