# Supplementary material for: Enhanced Room-Temperature Thermoelectric Performance of 2D-SnSe Alloys via Electric-Current-Assisted Sintering
Source: Materials (Basel). 2023 Jan 5;16(2):509. doi: 10.3390/ma16020509 (PMC9865124; doi:10.3390/ma16020509)
Supplement: Supplementary file 1 [file materials-16-00509-s001.zip › materials-2103653-supplementary.pdf]

Enhanced Room-Temperature Thermoelectric Performance of 2D-SnSe Alloys via Electric-Current-Assisted Sintering

Kesavan Manibalan *†, Meng-Yuan Ho, You-Cheng Du, Chen Hung Wei and Hsin-Jay Wu * †

Department of Materials Science and Engineering, National Yang Ming Chiao Tung University,
Hsinchu 30010, Taiwan

* Correspondence: kesavan.manibala@gmail.com (K.M.); ssky0211@nycu.edu.tw (H.J.W.)

† These authors contributed equally to this work.

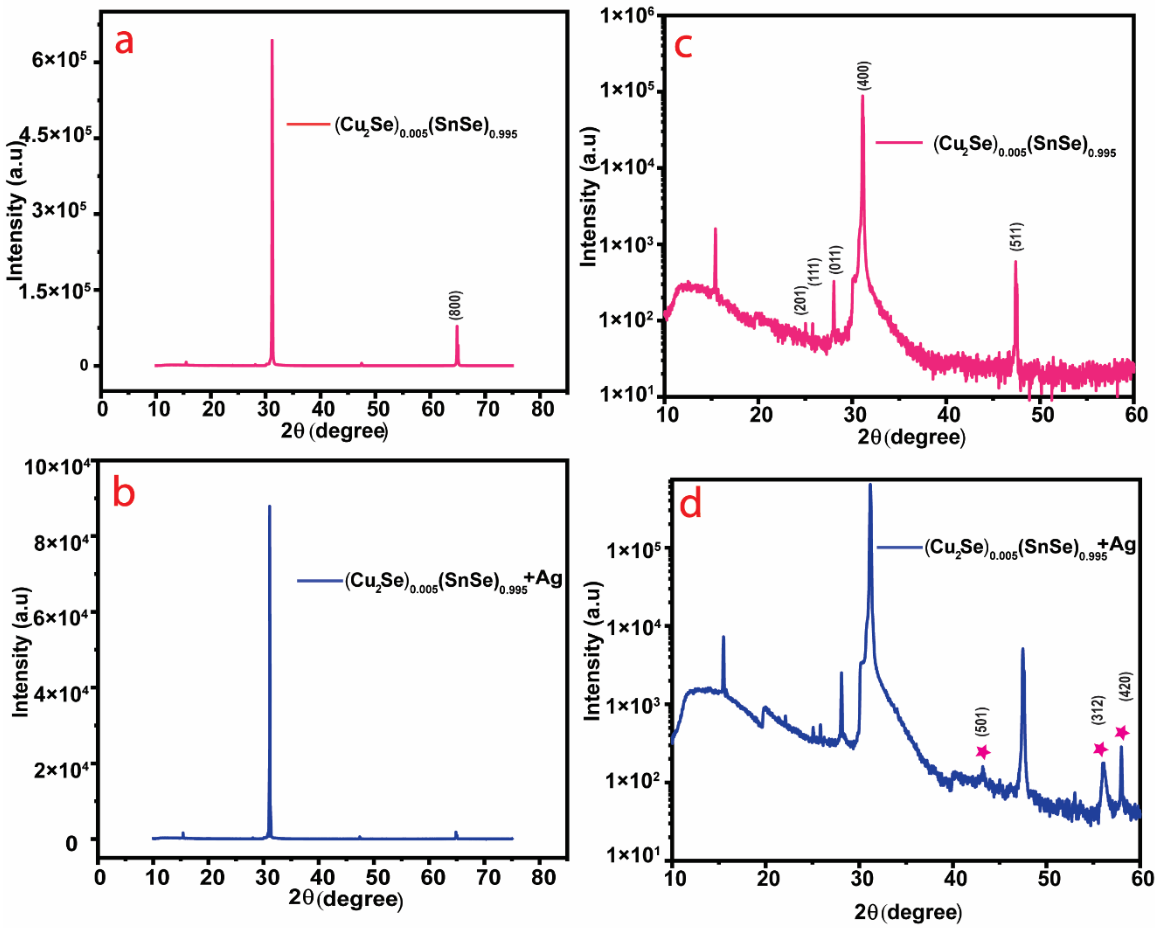

Figure S1. XRD analysis of $(Cu_2Se)_{0.005}(SnSe)_{0.995}$ alloy (a). XRD analysis of $(Cu_2Se)_{0.005}(SnSe)_{0.995}$ +Ag alloy (b). Corresponding stimulated pattern, respectively (c) and (d).

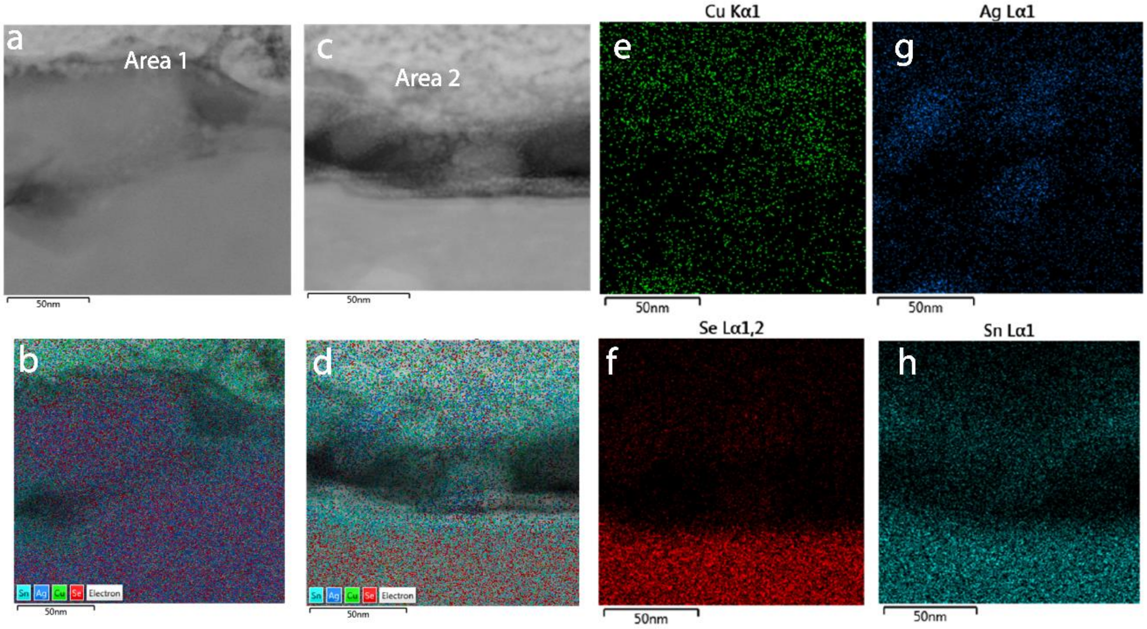

Figure S2. The TEM-EDS mapping of Ag element in the $(Cu_2Se)_{0.005}(SnSe)_{0.995}$+Ag alloy (Different area).

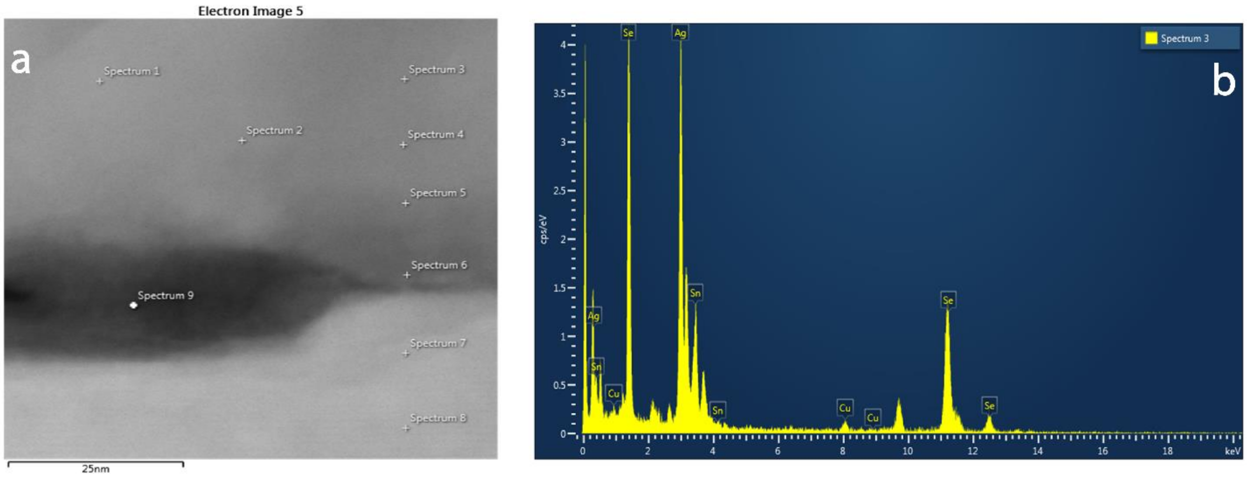

Figure S3. (A) TEM image of $(Cu_2Se)_{0.005}(SnSe)_{0.995}$+Ag alloy (B) EDS elemental analysis of $(Cu_2Se)_{0.005}(SnSe)_{0.995}$+Ag alloy, (C-F) TEM elemental mapping of $(Cu_2Se)_{0.005}(SnSe)_{0.995}$+Ag alloy.

Table S1. Reported method TE properties related to bilayer/interfacial layer based SnSe alloys in comparison to $(Cu_2Se)_{0.005}(SnSe)_{0.995}$+Ag alloy at 303 K.

Alloy	S (μV K^{-1})	σ (S m^{-1})	PF (mW m^{-1} K^{-2})	κ (W m^{-1} K^{-1})	Reference
**$(Cu_2Se)_{0.005}(SnSe)_{0.995}$+Ag**	**554**	**494**	**0.1487**	**1.43**	**This work**
SnSe + $Ag_{0.020}SnSe_2$	350	1600	0.0016	1.4	23
$Sn_{0.95}SeK_{0.05}$	400	-	0.08	1.1	24
$Sn_{0.93}Pb_{0.02}Se$	270	12	0.008	0.9	25
$Sn_{0.97}Ge_{0.03}Se$	220	13	0.001	1.0	26
6% Cl-doped $SnSe_2$/SnSe	200	5000	0.2	1.42	27
$Na_{0.005}Ag_{0.015}Sn_{0.98}Se$	215	47	0.23	1.18	28

References

23. Huo H. J; Zhang, Y; Guo, K; Yang, X.X; Xing, J.J; Lie, S; Zhang, J; Luo, J. Improvement of anisotropic thermoelectric performance in polycrystalline SnSe by metallic $AgSnSe_2$ compositing. *J. Alloys Compd*. **2022**, *908*,164649.
24. Gainza, J; Molto, S; Dura, F.S; O.J; Fernandez-Diaz, M.T; Biskup, N; Martinez, J.L; Alonso, J.A; Nemes, N.M. SnSe:Kx intermetallic thermoelectric polycrystals prepared by arc-melting. *J. Mater. Sci* **2022**, *57*, 8489–8503.
25. Tang, G; Liu, J; Zhang, J; Li,D; Rara, K.H; Xu, R; Lu, W; Liu, J; Zhang, Y.S; Feng, Z.Z. Realizing High Thermoelectric Performance below Phase Transition Temperature in Polycrystalline SnSe via Lattice Anharmonicity Strengthening and Strain Engineering. *ACS Appl. Mater. Interfaces*. **2018**, *10*, 30558–30565.
26. Chandra, S; Biswas, K. Realization of High Thermoelectric Figure of Merit in Solution Synthesized 2D SnSe Nanoplates via Ge Alloying. *J. Am. Chem. Soc*. **2019**, *57*, 8489–8503.
27. Shu, Y; Su, X; Xie, H; Zheng, G; Liu, W; Yan, Y; Luo, T.T; Yang, X; Yang, D.W; Uher, C; Tang, X.F. Modification of Bulk Heterojunction and Cl Doping for High-Performance Thermoelectric $SnSe_2$/SnSe Nanocomposites. *ACS Appl. Mater. Interfaces.* **2018**, *10*, 15793–15802.
28. Wang, S; Su, X; Bailey, T.P; Hu, T.Z; Zhang, Z; Tan, G; Yan, Y; Liu, W; Uher, C; Tang, X.F. Optimizing the average power factor of p-type (Na, Ag) co-doped polycrystalline SnSe. *RSC Adv*. **2019**, *9*, 7115–7122.
